# Supplementary material for: Integration of the Connectivity Map and Pathway Analysis to Predict Plant Extract’s Medicinal Properties—The Study Case of Sarcopoterium spinosum L
Source: Plants (Basel). 2022 Aug 24;11(17):2195. doi: 10.3390/plants11172195 (PMC9460920; doi:10.3390/plants11172195)
Supplement: Supplementary file 1 [file plants-11-02195-s001.zip › Supplementary-Figures.pdf]

Title: **Integration of the Connectivity Map and pathway analysis to predict plant extract's medicinal properties - the study case of *Sarcopoterium spinosum***

Authors: **Valid Gahramanov[1,] *, Moria Oz[3,] *, Tzemach Aouizerat[2], Mali Salmon-Divon[1], Tovit Rosenzweig[1], Jonathan Gorelick[2], Elyashiv Drori[2,3], Michael Y. Sherman[1], Bat Chen. R. Lubin[2,3] **.**

Supplementary Figure Legends

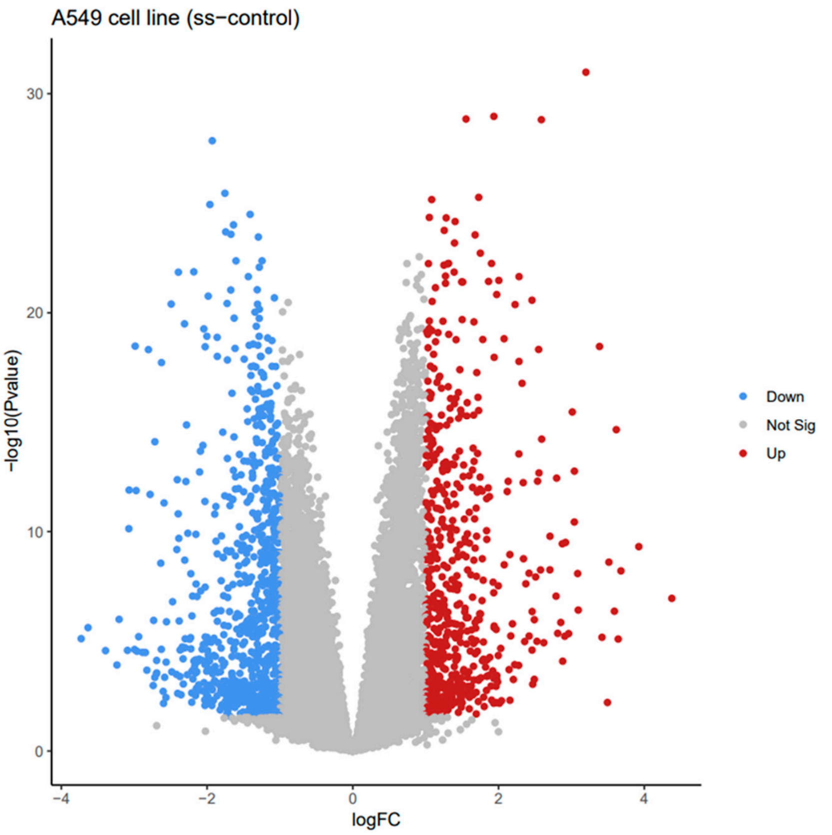

Figure S1: Volcano plot shows the differentially expressed genes after treatment of A549 lung cancer cells with plant extract. A plot is constructed by plotting the negative log of the p-value on the Y-axis (base 10). The X-axis is the log of the fold change between two conditions – treated and untreated. Log2F=1, and p-value less than 0.05 has been set up as a significant threshold. The genes that have a large magnitude of the fold change, as well as high statistical significance, appear

towards the top of the plot. Blue – indicates downregulated, red – upregulated genes, gray – includes the information of the genes that have no significant changes between two conditions.

Table S1: The entire list of differentially expressed genes after treatment of A549 cells with SSE extract. (Shown in excel file)

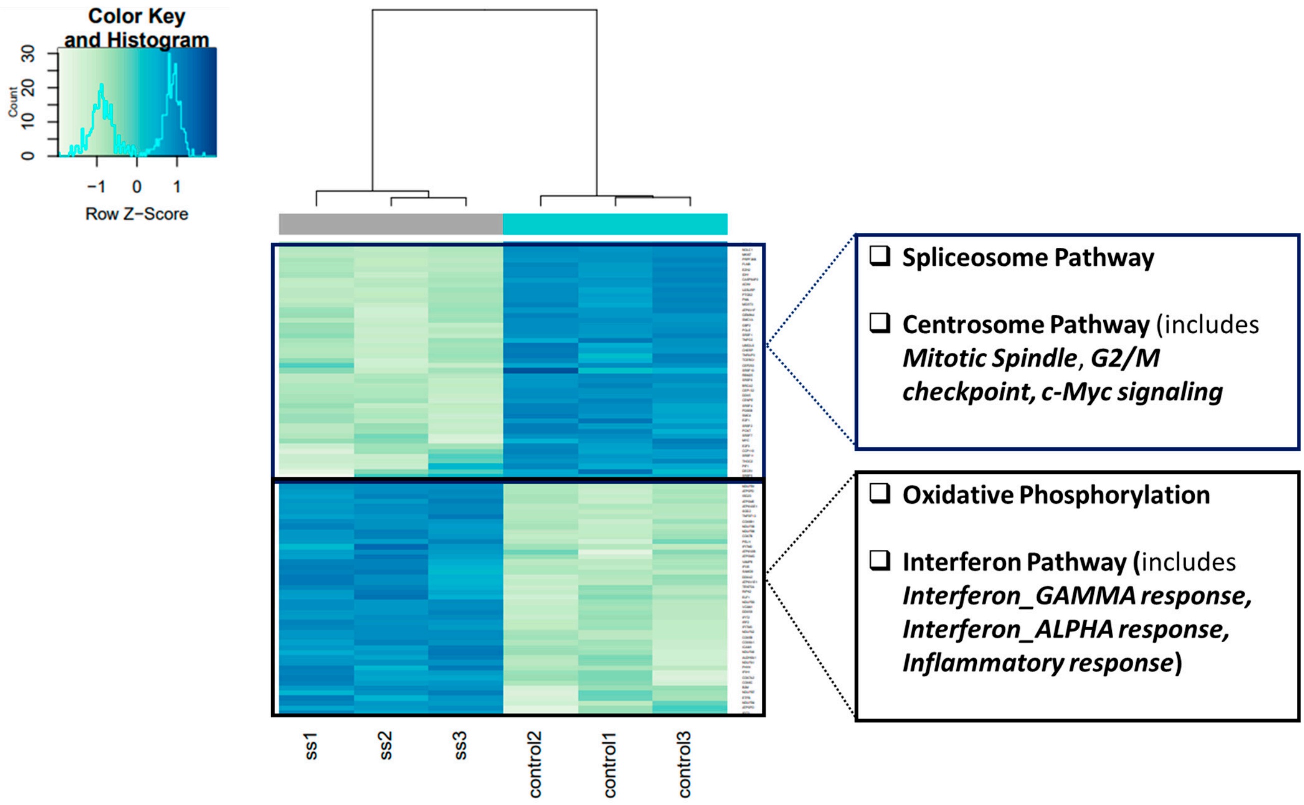

Figure S2: Heatmap of the top 100 DEGs (differentially expressed genes) from chosen pathways. Genes upregulated are in blue. Genes downregulated are in green. The differences are set as FC>1 and adjusted P-value <0.05.

Table S2: The list of the pathways (both upregulated and downregulated) that have been obtained after GSEA analysis on A549 treated cells (Gene Set Enrichment Analysis). FDR<0.250 cutoffs for the GSEA analysis have been used. (Shown in excel file)

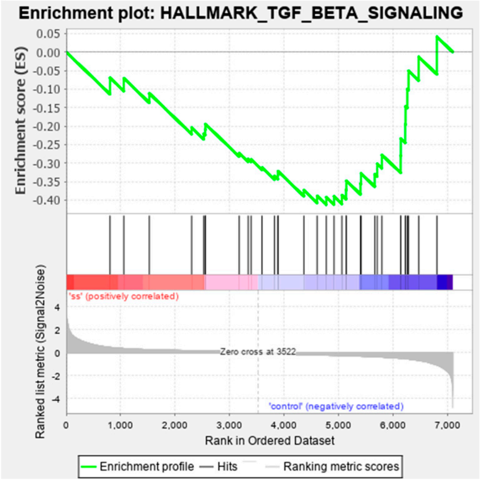
Enrichment plot: HALLMARK_TGF_BETA_SIGNALING
Enrichment score (ES)
'ss' (positively correlated)
Zero cross at 3522
'control' (negatively correlated)
Ranked list metric (Signal2Noise)
Rank in Ordered Dataset
Enrichment profile — Hits — Ranking metric scores

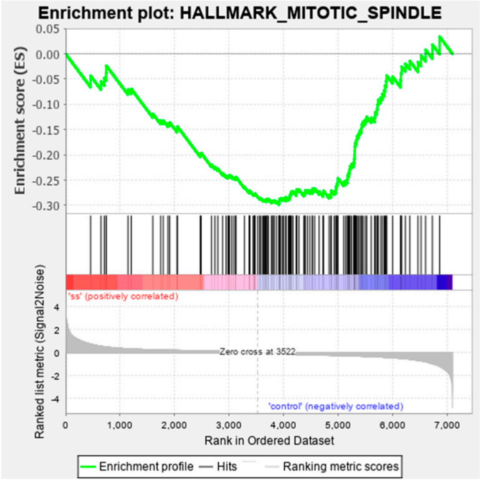
Enrichment plot: HALLMARK_MITOTIC_SPINDLE
Enrichment score (ES)
'ss' (positively correlated)
Zero cross at 3522
'control' (negatively correlated)
Ranked list metric (Signal2Noise)
Rank in Ordered Dataset
Enrichment profile — Hits — Ranking metric scores

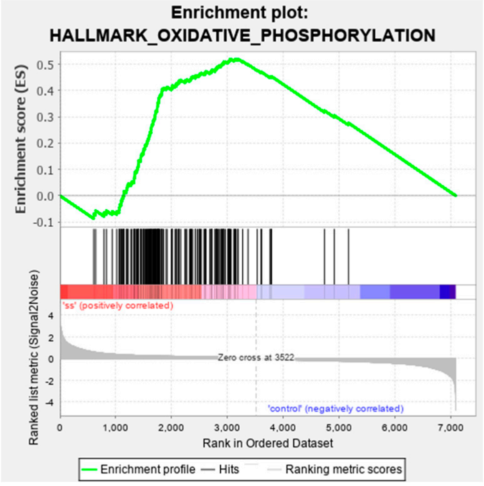
Enrichment plot:
HALLMARK_OXIDATIVE_PHOSPHORYLATION
Enrichment score (ES)
'ss' (positively correlated)
Zero cross at 3522
'control' (negatively correlated)
Ranked list metric (Signal2Noise)
Rank in Ordered Dataset
Enrichment profile — Hits — Ranking metric scores

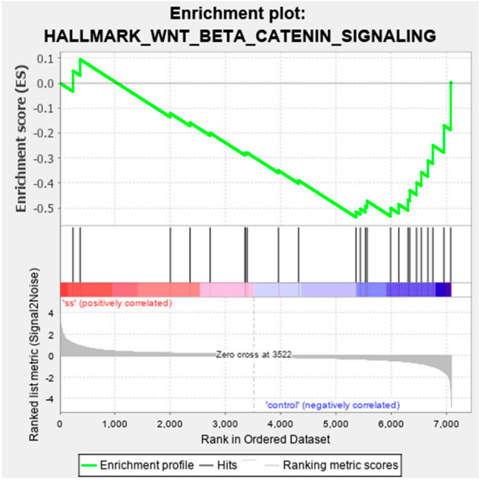
Enrichment plot:
HALLMARK_WNT_BETA_CATENIN_SIGNALING
Enrichment score (ES)
'ss' (positively correlated)
Zero cross at 3522
'control' (negatively correlated)
Ranked list metric (Signal2Noise)
Rank in Ordered Dataset
Enrichment profile — Hits — Ranking metric scores

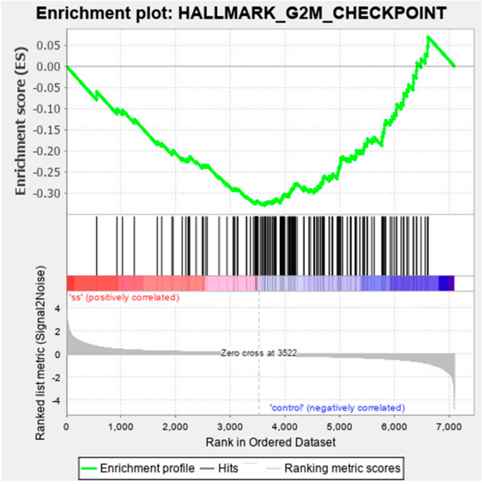
Enrichment plot: HALLMARK_G2M_CHECKPOINT
Enrichment score (ES)
'ss' (positively correlated)
Zero cross at 3522
'control' (negatively correlated)
Ranked list metric (Signal2Noise)
Rank in Ordered Dataset
Enrichment profile — Hits — Ranking metric scores

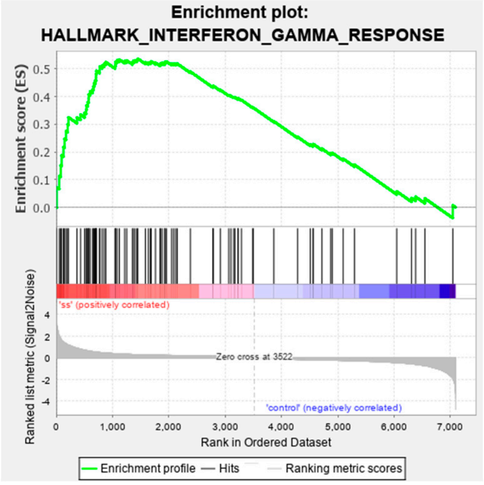
Enrichment plot:
HALLMARK_INTERFERON_GAMMA_RESPONSE
Enrichment score (ES)
'ss' (positively correlated)
Zero cross at 3522
'control' (negatively correlated)
Ranked list metric (Signal2Noise)
Rank in Ordered Dataset
Enrichment profile — Hits — Ranking metric scores

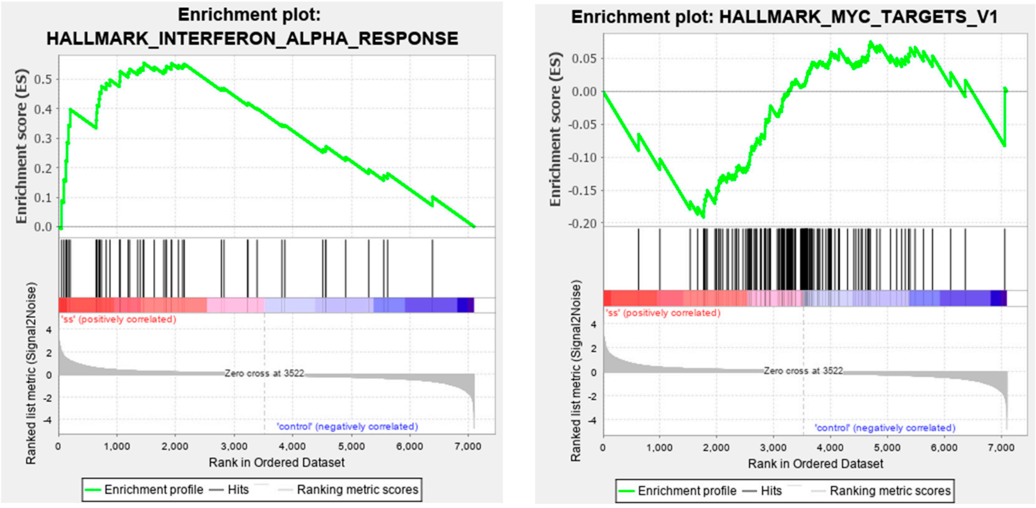

Figure S3: GSEA analysis of extract effect on A549 lung cancer cell line. Gene set enrichment analysis (GSEA) was performed with the canonical pathway, or biological process, gene sets in GSEA Molecular Signatures Database. The green curve corresponds to the ES (enrichment score). The normalized enrichment score (NES) and the corresponding P-value are reported within each graph. The graphs on the top panels represent upregulated pathways, whereas graphs on the bottom panel represent downregulated pathways. In each graph genes on the far left (red) correlated with the most upregulated genes in the extract treatment and genes on the far right (blue) correlated with the most downregulated genes in the treatment. In each graph, vertical black lines indicate the position of each gene in the gene set.

Table S3: The list of the pathways (both upregulated and downregulated) that have been obtained after GSEA analysis on Mouse adipocyte 3T3 treated cells (Gene Set Enrichment Analysis). FDR<0.250 cutoffs for the GSEA analysis have been used. (Shown in excel file)

Table S4: Representation of the signaling pathways of the drug compounds obtained from GSEA-CMAP integration analysis.
