# Supplementary figures and images for: Integration of the Connectivity Map and Pathway Analysis to Predict Plant Extract’s Medicinal Properties—The Study Case of Sarcopoterium spinosum L
Source: Plants (Basel). 2022 Aug 24;11(17):2195. doi: 10.3390/plants11172195 (PMC9460920; doi:10.3390/plants11172195)

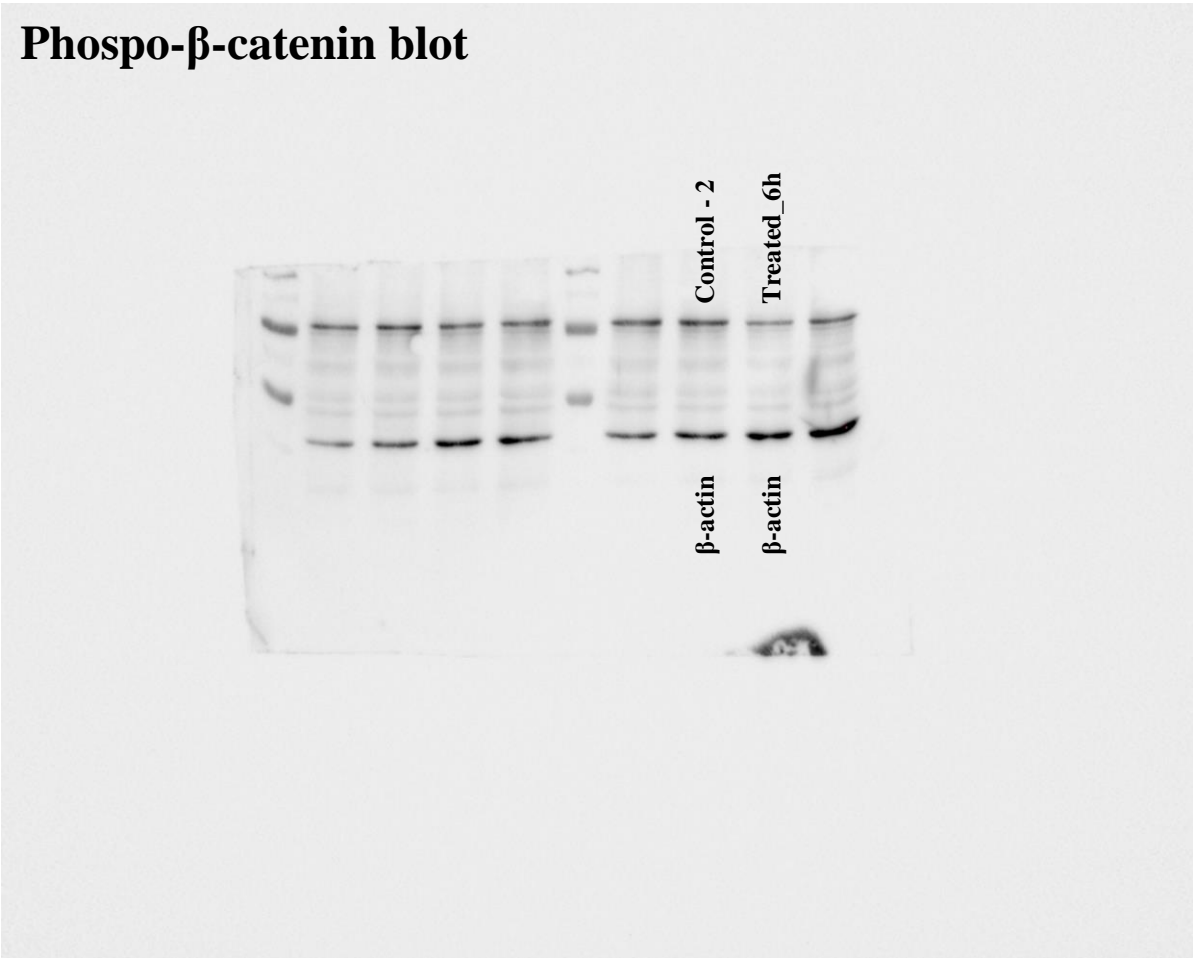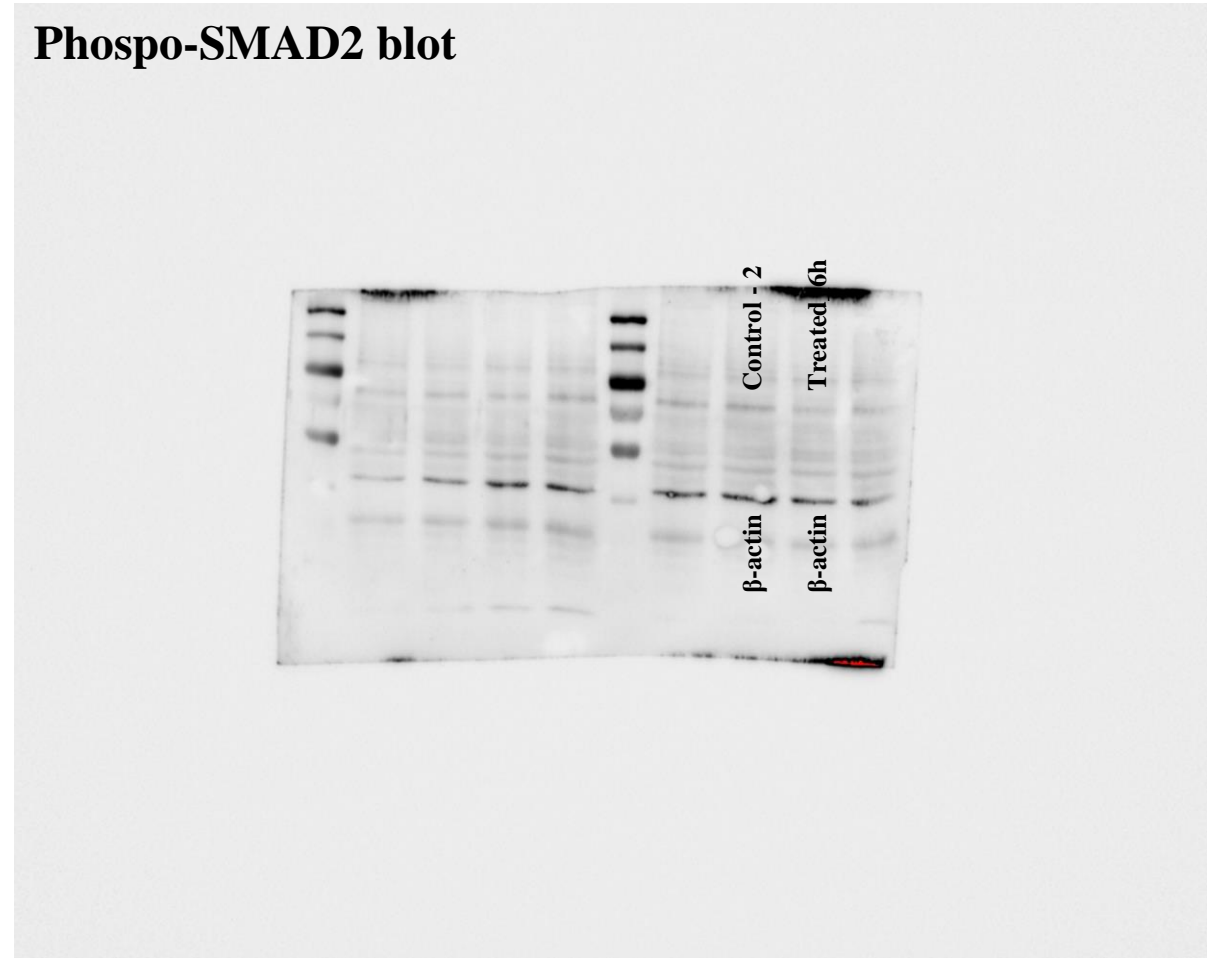

LC3\_1 and LC3\_2 blot

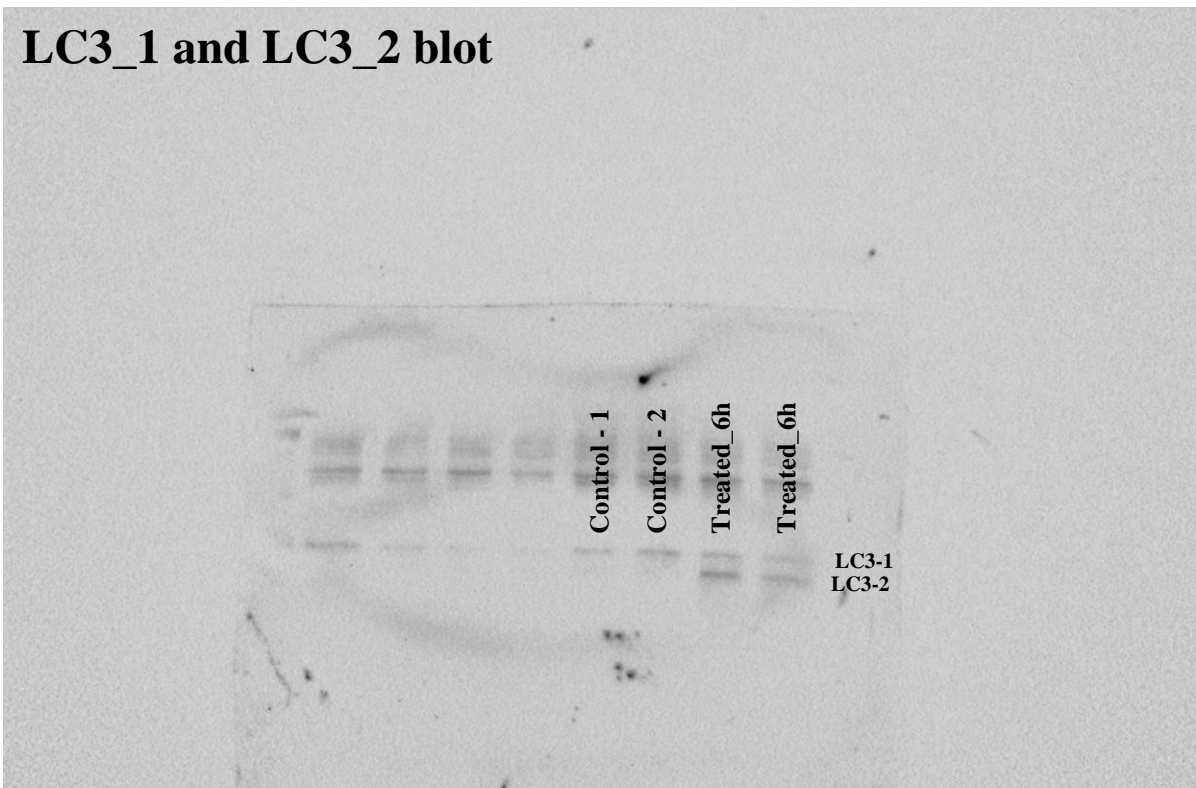

LC3\_1 and LC3\_2  $\beta$ -actin blot

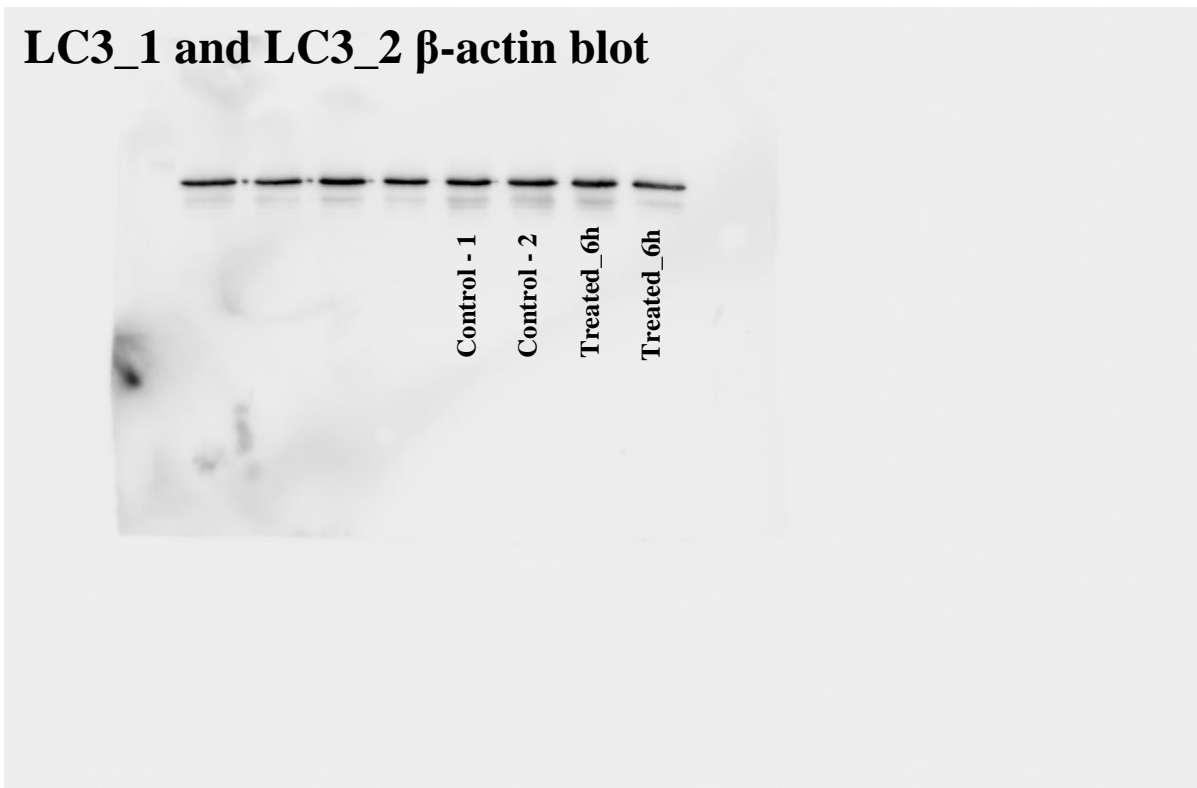

Supplement: Supplementary file 1 [file plants-11-02195-s001.zip › Original-blots.pdf]
